# YC-1 Prevents Tumor-Associated Tissue Factor Expression and Procoagulant Activity in Hypoxic Conditions by Inhibiting p38/NF-κB Signaling Pathway

**DOI:** 10.3390/ijms20020244

**Published:** 2019-01-09

**Authors:** Kan-Yen Hsieh, Chien-Kei Wei, Chin-Chung Wu

**Affiliations:** 1 Graduate Institute of Natural Products, Kaohsiung Medical University, Kaohsiung 80708, Taiwan; u101831004@kmu.edu.tw (K.-Y.H.); weichankai@yahoo.com.tw (C.-K.W.); 2 Research Center for Natural Product and Drug Development, Kaohsiung Medical University, Kaohsiung 80708, Taiwan; 3 Department of Medical Research, Kaohsiung Medical University Hospital, Kaohsiung 80708, Taiwan

**Keywords:** tissue factor, hypoxia, cancer, procoagulant activity, MAP kinases

## Abstract

Tissue factor (TF) expressed in cancer cells has been linked to tumor-associated thrombosis, a major cause of mortality in malignancy. Hypoxia is a common feature of solid tumors and can upregulate TF. In this study, the effect of YC-1, a putative inhibitor of hypoxia-inducible factor-1α (HIF-1α), on hypoxia-induced TF expression was investigated in human lung cancer A549 cells. YC-1 selectively prevented hypoxia-induced TF expression and procoagulant activity without affecting the basal TF levels. Surprisingly, knockdown or pharmacological inhibition of HIF-1α failed to mimic YC-1′s effect on TF expression, suggesting other mechanisms are involved. NF-κB, a transcription factor for TF, and its upstream regulator p38, were activated by hypoxia exposure. Treatment of hypoxic A549 cells with YC-1 prevented the activation of both NF-κB and p38. Inhibition of p38 suppressed hypoxia-activated NF-κB, and inhibited TF expression and activity to similar levels as treatment with an NF-κB inhibitor. Furthermore, stimulation of p38 by anisomycin reversed the effects of YC-1. Taken together, our results suggest that YC-1 prevents hypoxia-induced TF in cancer cells by inhibiting the p38/NF-κB pathway, this is distinct from the conventional anticoagulants that systemically inhibit blood coagulation and may shed new light on approaches to treat tumor-associated thrombosis.

## 1. Introduction

Venous thromboembolism (VTE) is a major complication and cause of mortality in patients with cancer. Up to 20% of all cancer patients are diagnosed with deep venous thrombosis or pulmonary embolism [1]. Among the different solid tumor types, lung cancer patients have the highest incidence rate of VTE. Multiple factors, including chemotherapy and molecular target therapy, can contribute to cancer-associated VTE, but the increased procoagulant activity of cancer cells is generally considered to be the major cause [2].

Tissue factor (TF), an integral membrane protein, is the primary cellular initiator of blood coagulation [3]. Upon a vascular injury, TF is exposed to circulating factor VIIa, forming a complex that activates factor X. This, in turn, converts prothrombin into thrombin and results in blood clot formation. TF has been found overexpressed in many types of cancer cells and accounts for cancer procoagulant activity [4,5]. Therefore, cancer cell-expressed TF is considered to be the trigger of VTE [6]. Moreover, clinical data reveal that increased TF expression was associated with reduced survival of patients with non–small cell lung cancer [7]. Currently, heparins (unfractionated or low-molecular-weight) are the main anticoagulants for treating cancer-associated VTE; however, they suffer from some limitations, including increased risk of bleeding complications and recurrence of VTE after discontinuing anticoagulation [8]. It is worth noting that bleeding risk can be six times higher under anticoagulation for VTE in patients with cancer than in patients without cancer [9]. Therefore, the treatment of cancer-associated VTE is still a challenge for clinicians.

Hypoxia is a common feature of solid tumors. To survive and grow in a hypoxic microenvironment, cancer cells must undergo a variety of adaptive changes, including metabolic adaptation, increased resistance to apoptosis, and promotion of angiogenesis and metastasis [10]. In addition, hypoxia-induced upregulation of TF and procoagulant activity has been reported in a variety of cancer types, including, breast cancer [11], ovarian cancer [12], glioma cancer [13], and lung cancer [14]. A major mechanism mediating adaptive responses to hypoxia is the regulation of transcription by hypoxia-inducible factor-1α (HIF-1α) [15]. Under normoxic conditions, HIF-1α is hydroxylated by prolyl-hydroxylases and degraded by the ubiquitin-proteasome pathway. However, under hypoxic conditions, HIF-1α is stabilized and mediates transcriptional activation of genes involved in energy metabolism, apoptosis, angiogenesis and metastasis [16]. Because hypoxia is nearly exclusively restricted to cancer cells, hypoxia-targeted therapy may be an attractive strategy for treating solid cancers, including lung cancer [17].

YC-1 [3-(5′-hydroxymethyl-2′-furyl)-1-benzylindazole], a synthetic compound, was firstly reported as a nitric oxide-independent activator of soluble guanylyl cyclase (sGC) in platelets and exhibited in vitro and in vivo antiplatelet activity through elevation of intracellular cGMP levels [18,19,20]. In recent years, YC-1 has emerged as potential hypoxia-targeted agent because of its inhibitory effect on HIF-1α [21], which is crucial for tumor angiogenesis under hypoxic microenvironment. Notably, YC-1 prevented hypoxia-induced HIF-1α accumulation in a sGC/cGMP-independent manner [22]. In xenograft models of different human cancers, such as hepatoma, cervical carcinoma, and lung cancer, YC-1 significantly inhibited tumor growth and prolonged the survival periods, associating with reduced HIF-1α expression and blocked angiogenesis [23,24]. YC-1 was reported to enhance the antitumor effect of radiation on hypoxic lung cancer cells [25]. Furthermore, YC-1 showed anti-proliferative [26] and anti-invasion/anti-metastatic activity in several in vitro and in vivo cancer models [27,28,29]. Importantly, no serious toxicity was observed in mice during treatment with YC-1 [23,25].

Hypoxia-targeted strategies for cancer therapy have been intensively explored; however, much less attention has been focused on specific inhibition of hypercoagulability in hypoxic cancer. Therefore, in the present study, we aim to investigate the effects and the underlying mechanisms of YC-1 in preventing hypoxia-induced TF expression and procoagulant activity in human lung cancer A549 cells.

## 2. Results

### 2.1. YC-1 Inhibits Hypoxia-Induced TF Expression in Human Cancer Cells

**Figure 1:**
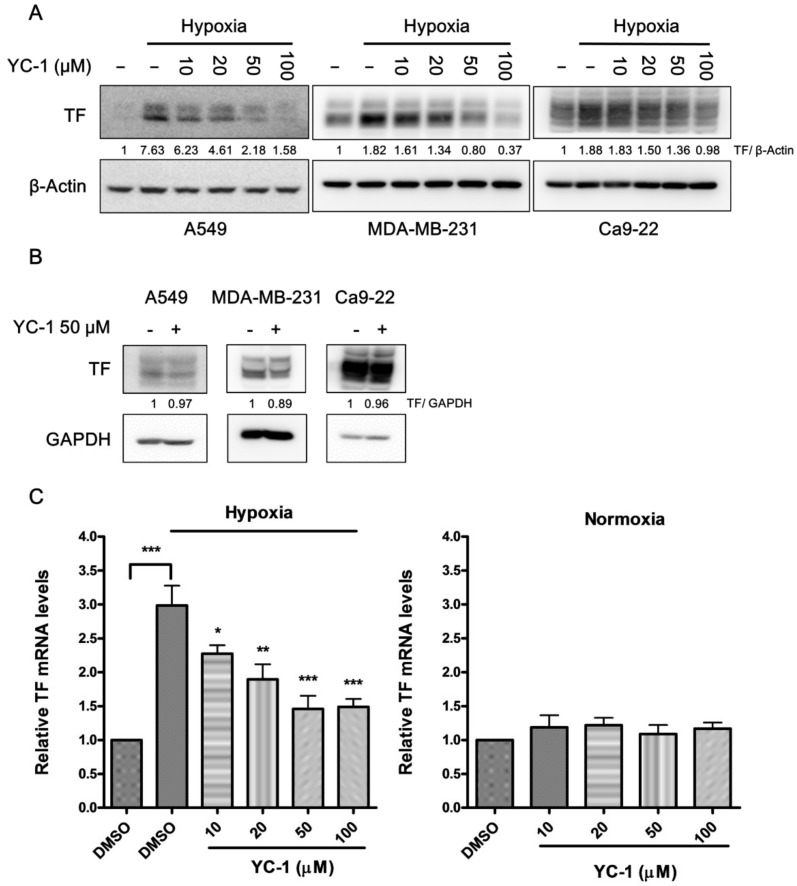
YC-1 inhibits hypoxia-induced TF expression in human cancer cell lines. Human lung cancer A549, breast cancer MDA-MB-231, and oral cancer Ca9-22 cells were pretreated with DMSO (vehicle control) or YC-1 (10–100 μM) for 1 h, and then incubated under hypoxic (**A**) or normoxic (**B**) conditions for 24 h. The protein expression of TF was evaluated by Western blotting. (**C**) A549 cells were pretreated with DMSO or YC-1 and exposed to hypoxia or normoxia for 4 h. The mRNA levels of TF were determined by real-time PCR. All results are presented as mean ± SEM (*n* = 3). * = *p* < 0.05, ** = *p* < 0.01, *** = *p* < 0.001.

In the three kinds of human cancer cell lines-lung cancer A549 cells, breast cancer MDA-MB-231 cells, and oral cancer Ca9-22 cells, the protein levels of TF were significantly increased by hypoxia exposure for 24 h (Figure 1A). This phenomenon was most pronounced in A549 cancer cells that expressed very low levels of TF under normoxic conditions. Pretreatment of cancer cells with YC-1 (10–100 μM) led to inhibition of hypoxia-induced TF in a dose-dependent manner. In contrast, YC-1 had no significant effect on TF expression under normoxic conditions (Figure 1B).

We next examined the mRNA levels of TF in A549 cancer cells by using real-time RT-PCR. Figure 1C shows that hypoxia caused a 3-fold increase in TF mRNA. In the same concentration range (10–100 μM) used for inhibition of TF protein expression, YC-1 also prevented hypoxia-induced increase in TF mRNA and showed no effect on the basal mRNA levels in normoxic condition. These results suggested that YC-1 inhibited TF expression at the transcription level in hypoxic condition.

The cytotoxic effect of YC-1 on A549 cancer cells was examined by MTT assay. Treatment of A549 cells with YC-1 (10–100 μM) under hypoxic or normoxic conditions for 24 h reduced the cell viability by up to 22.8 or 33.4%, respectively (Supplementary Figure S1). Therefore, the inhibitory effect of YC-1 on hypoxia-induced TF is unlikely due to its cytotoxicity.

Because A549 cancer cells presented most evident TF expression in response to hypoxia, the cancer cell line was chosen for further study on the molecular mechanism underlying YC-1 inhibition of hypoxia-induced TF.

### 2.2. YC-1 Inhibits Hypoxia-Enhanced Procoagulant and Platelet-Stimulating Activity in A549 Cells

**Figure 2:**
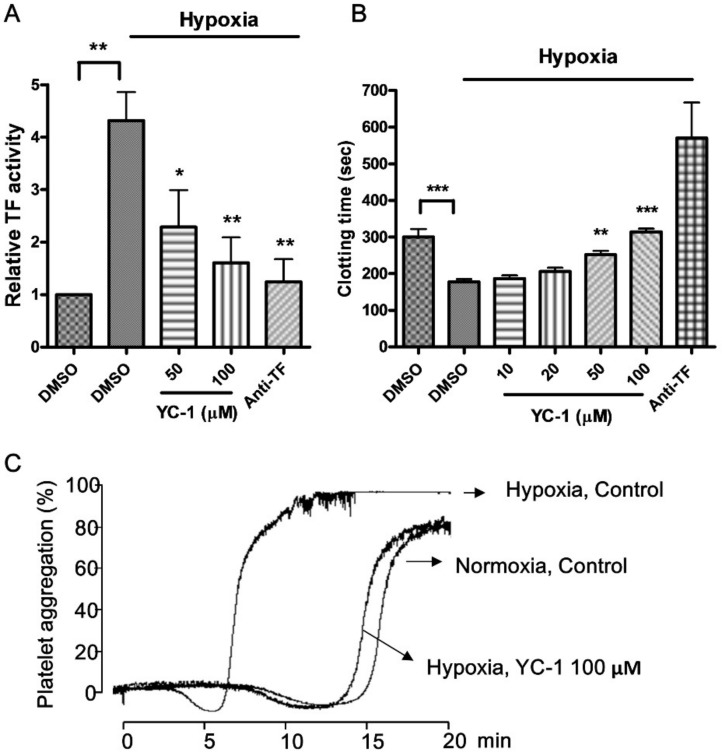
YC-1 reduces hypoxia-induced TF procoagulant activity in A549 cells. A549 cells were pretreated with DMSO or YC-1 for 1 h, and then incubated under hypoxic or normoxic conditions for 24 h. (**A**) The cell surface TF activity was measured by a coupled amidolytic assay of TF-dependent factor Xa generation; (**B**) Cancer cell-induced plasma clotting was determined by tilt tube assay. Anti-TF antibody (20 μg/mL) was used as positive control. (**C**) A549 cells treated with DMSO or YC-1 (100 μM) in normoxia or hypoxia were harvested (1 × 10^5^ cells/mL) and mixed with platelet suspension (3 × 10^8^ platelets/mL). Platelet aggregation was induced by adding human plasma (0.25%). All results are presented as mean ± SEM (*n* = 3). * = *p* < 0.05, ** = *p* <0.01, *** = *p* < 0.001.

In order to confirm if TF’s function was enhanced in parallel with TF expression in hypoxic A549 cancer cells, the cell surface TF procoagulant activity was measured by a coupled amidolytic assay of factor Xa generation. As shown in Figure 2A, hypoxia exposure enhanced TF procoagulant activity by 4.3-fold compared to that in normoxic conditions, and this increase was abolished by anti-TF antibody. Pretreatment of A549 cancer cells with YC-1 resulted in almost complete inhibition of hypoxia-induced TF activity.

The procoagulant activity of TF in hypoxia-treated cancer cells was further studied by using plasma clotting and platelet aggregation assays [13,30], both are more physiologically relevant than the amidolytic assay. In plasma clotting assay, the clotting time in the presence of hypoxia-treated A549 cancer cells was significantly shorter than that under normoxic conditions, and this phenomenon was reversed by adding anti-TF antibody. Treatment of A549 cancer cells with YC-1 (10–100 μM) prior to hypoxia exposure significantly prolonged plasma clotting time to near that observed in the normoxic control group, indicating that hypoxia-induced TF activity was inhibited by YC-1 (Figure 2B). A similar effect of YC-1 was also seen in platelet aggregation assay (Figure 2C). In the presence of plasma and normoxic A549 cancer cells, human platelets were slowly activated and aggregated, this process took 15–20 min. In contrast, hypoxic A549 cancer cells were able to induce platelet aggregation within 5–10 min. The cancer cell-induced platelet aggregation was dependent on TF since it was abolished by anti-TF antibody (data not shown). Pretreatment of hypoxic cancer cells with YC-1 led to reduction in their platelet-stimulating activity. Of note, YC-1 treatment did not affect normoxic A549 cell-induced plasma clotting and platelet aggregation (Figure S2). These results suggest that YC-1 selectively inhibits hypoxia-enhanced procoagulant activity in A549 cells.

### 2.3. YC-1 Inhibits Hypoxia-Induced TF Via a HIF-1α-Independent Manner in A549 Cells

**Figure 3:**
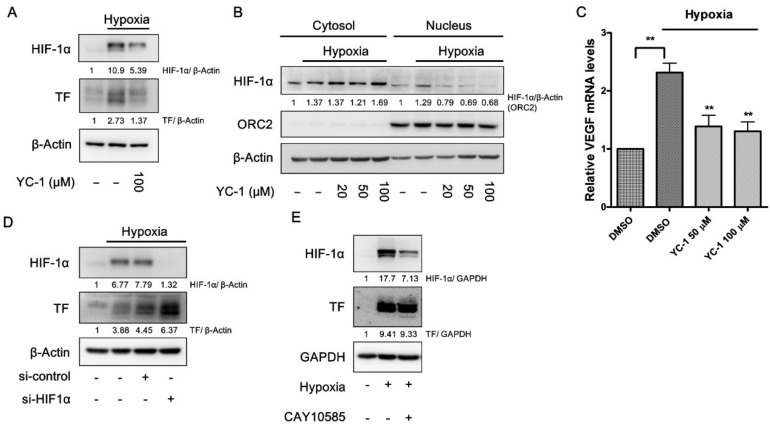
YC-1 inhibits hypoxia-induced TF via a HIF-1α-independent manner in A549 cells. (**A**,**B**) YC-1 inhibits HIF-1α accumulations and decreases HIF-1α nuclear translocation in response to hypoxia. A549 cancer cells were treated with DMSO or YC-1 for 1 h and incubated under normoxia or hypoxia for 24 h. Whole-cell lysates (**A**) and the nuclear fractions (**B**) were subjected to Western blotting for HIF-1α; In (**B**), ORC2 was used as a loading control in the nuclear fractions; (**C**) YC-1 inhibits hypoxia-induced upregulation of VEGF. A549 cancer cells pretreated with DMSO or YC-1 were exposed to hypoxia for 4 h. The mRNA levels were determined by real-time PCR. All results were presented as mean ± SEM (*n* = 3). ** = *p* < 0.01. Knockdown (**D**) or pharmacological inhibition (**E**) of HIF-1α fails to prevent hypoxia-induced TF expression. A549 cancer cells were transiently transfected with si-HIF-1α (50 nM) or negative control siRNA (**D**), or treated with the HIF-1α inhibitor CAY10585 (10 μM) for 1 h (**E**), followed by exposure to hypoxia for 24 h. The protein levels of TF in the cell lysates were determined by Western blotting.

In A549 cells, YC-1 prevented the accumulation of HIF-1α in response to hypoxia (Figure 3A). Consistently, the nuclear levels of HIF-1α, as well as the mRNA expression of the HIF-1α target gene vascular endothelial growth factor (VEGF), were also prevented by YC-1 (Figure 3B,C). However, knockdown of HIF-1α expression with siRNA enhanced, rather than inhibited, hypoxia-induced TF (Figure 3D). Furthermore, CAY10585, a putative HIF-1α inhibitor with a structure unrelated to YC-1, distinctly suppressed HIF-1α accumulation in hypoxia. However, the inhibition of HIF-1α failed to mimic YC-1′s effect on TF expression under hypoxic conditions (Figure 3E).

### 2.4. YC-1 Activates Cyclic Nucleotide-Dependent Protein Kinases in A549 Cells

**Figure 4:**
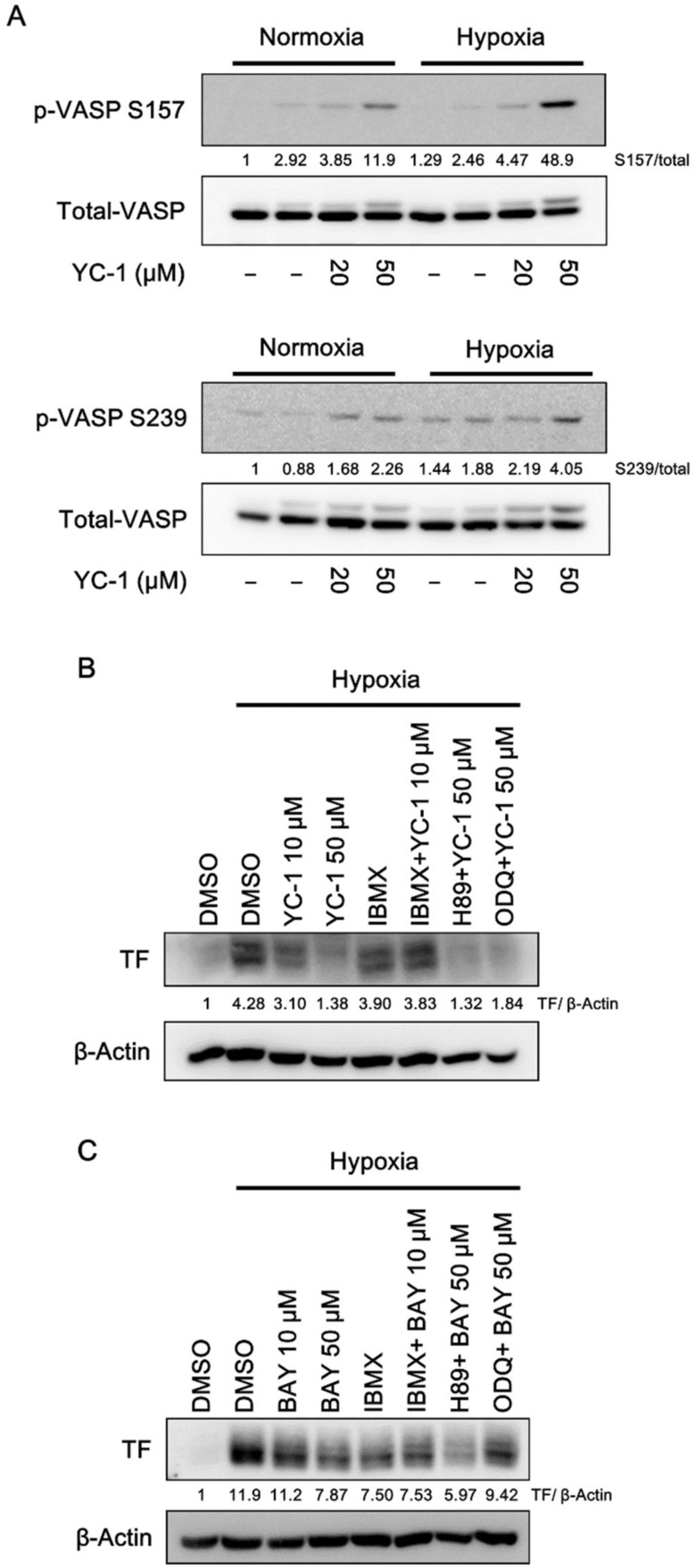
YC-1 activates cyclic nucleotide-dependent protein kinases in A549 cells. (**A**) A549 cells were pretreated with YC-1 for 1 h and incubated under normoxia or hypoxia for another 1 h. Cell lysates were subjected to Western blotting for phospho-VASP. (**B**,**C**) A549 cells were pretreated with the PDE inhibitor IBMX (100 μM), the sGC inhibitor ODQ (10 μM), or the PKA inhibitor H89 (5 μM) for 30 min, then treated with YC-1 or BAY 41-2272 (BAY) for 1 h and incubated under hypoxia for another 24 h. TF expression was determined by Western blotting.

In our previous studies, YC-1 has been demonstrated as a nitric oxide-independent sGC activator, which directly caused an elevation of intracellular cGMP levels, and also enhanced cAMP levels in an indirect manner in human platelets [19]. Therefore, we would like to investigate if YC-1 exerts its anti-TF effect through cyclic nucleotides. The phosphorylation of vasodilator-stimulated phosphoprotein (VASP) at Ser239 or Ser157 was measured as a marker of activation of Protein kinase G (PKG) or Protein kinase A (PKA). As shown in Figure 4A, YC-1 induced VASP phosphorylation at Ser157 and Ser239 under both normoxic and hypoxic conditions, indicating that YC-1 was able to activate cGMP-dependent and/or cAMP-dependent pathways in A549 cancer cells. However, neither ODQ (a sGC inhibitor) nor H89 (a PKA inhibitor) reversed YC-1 inhibition of hypoxia-induced TF (Figure 4B). Furthermore, the non-selective cyclic nucleotide phosphodiesterase (PDE) inhibitor, IBMX, also failed to enhance the action of YC-1 (Figure 4B). BAY 41-2272, which is a more potent sGC activator than YC-1 [31], showed a similar inhibitory effect on hypoxia-induced TF; whereas its effect could be enhanced by IBMX, and reversed by ODQ (Figure 4C). These results suggested that other cyclic nucleotides-independent mechanisms may contribute to YC-1′s action on hypoxia-induced TF expression.

### 2.5. YC-1 Inhibits Hypoxia-Induced NF-κB Activation

**Figure 5:**
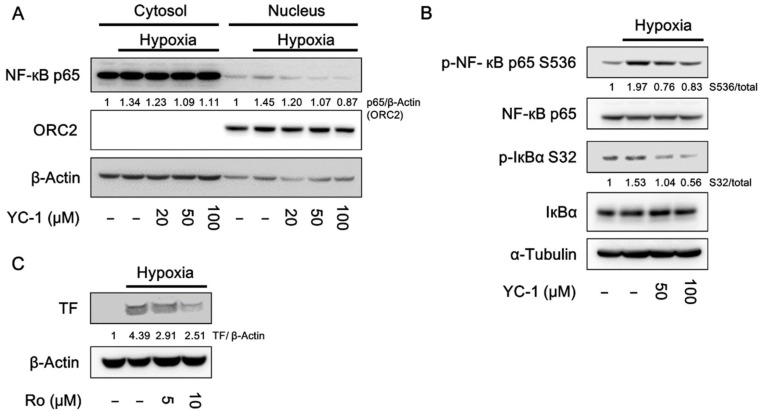
YC-1 inhibits hypoxia-induced NF-κB activation in A549 cells. A549 cancer cells were pretreated with YC-1 for 1 h and exposed to normoxia or hypoxia. Protein levels of NF-κB p65 in the cytosolic and nuclear fractions (**A**) and the phosphorylation of NF-κB p65 and IκBα in the whole-cell lysates (**B**) were determined by Western blotting. (**C**) A549 cells were pretreated with the NF-κB inhibitor RO 106-9920 for 1 h and exposed to hypoxia for 24 h. TF expression was determined by Western blotting.

The transcription factor NF-κB has been reported to mediate TF expression in response to hypoxia [32]. Because YC-1 inhibited hypoxia-induced TF expression at the transcription level, we would like to examine the effect of YC-1 on NF-κB signaling. As shown in Figure 5A, A549 cancer cells exposed to hypoxia resulted in nuclear translocation of NF-κB p65 subunit, and this event was inhibited by YC-1. In contrast, YC-1 failed to inhibit nuclear translocation of SP1 and early growth response 1 (EGR1), which are also transcription factors responsible for TF expression (Figure S3). Consistent with the result of NF-κB nuclear translocation, YC-1 also inhibited hypoxia-induced phosphorylation of p65 (Figure 5B). Moreover, the NF-κB inhibitor Ro 106-9920 mimicked the effect of YC-1 on inhibiting hypoxia-induced TF expression (Figure 5C). These results suggested that inhibition of NF-κB signaling by YC-1 may contributes to negative regulation of TF expression.

### 2.6. YC-1 Prevents Hypoxia-Induced TF through Inhibition of the p38/NF-κB Pathway

**Figure 6:**
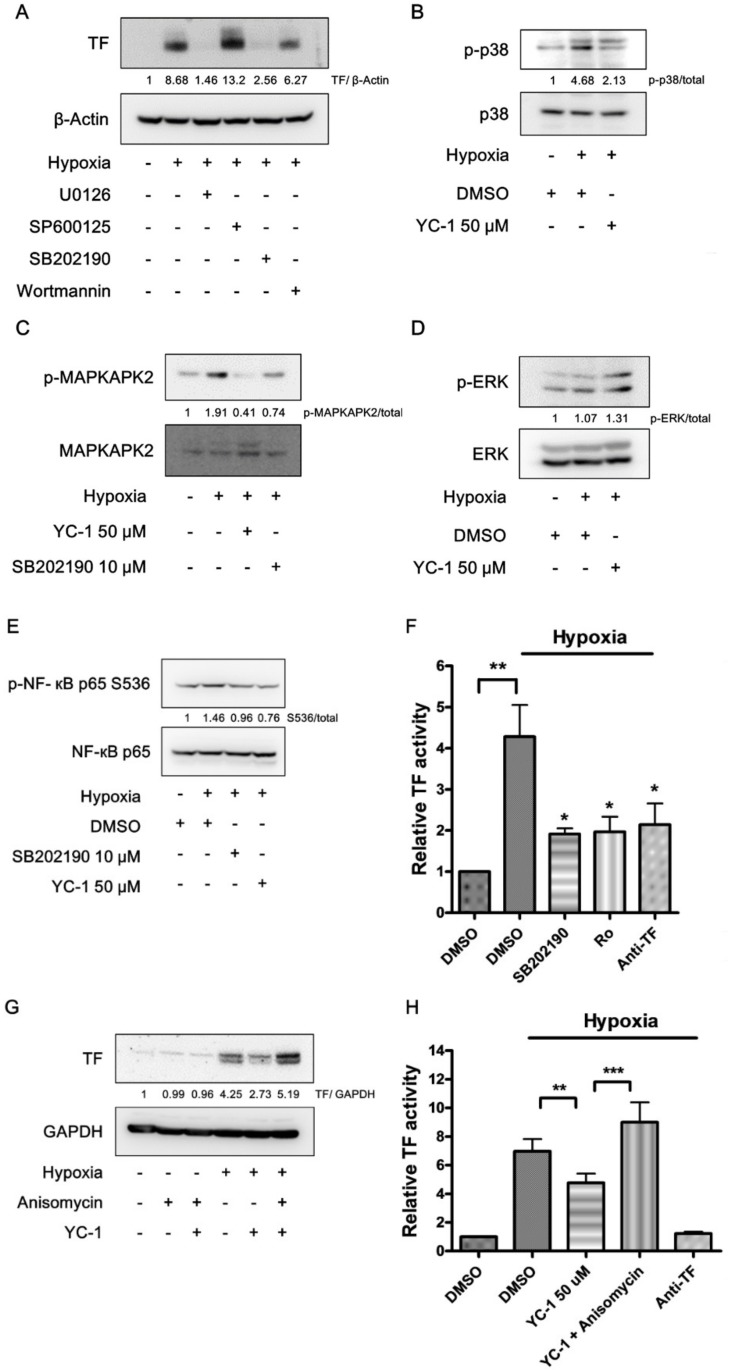
YC-1 prevents hypoxia-induced TF through inhibition of the p38/NF-κB pathway. (**A**) Effects of specific inhibitors of MAPKs and Akt on hypoxia-induced TF expression. A549 cells were treated with U0126 (10 μM), SP600125 (10 μM), SB202190 (10 μM), and wortmannin (0.1 μM) for 1 h, and exposed to hypoxia for 24 h. Cell lysates were subjected to Western blotting for TF. (**B**,**C**) YC-1 inhibits hypoxia-stimulated p38 activation. Cells pretreated with YC-1 were exposed to hypoxia for 15 min (**B**) or 2 h (**C**). Cell lysates were subjected to Western blotting for p38 and MAPKAPK2. (**D**) The effect of YC-1 on ERK activation in hypoxic conditions. A549 cells were treated as in (**B**), and the cell lysates were subjected to Western blotting for ERK. (**E**) The p38 inhibitor prevents hypoxia-induced NF-κB activation. A549 cells treated with SB202190 (10 μM) or YC-1 (50 μM) were exposed to hypoxia for 2 h. Cell lysates were subjected to Western blotting for NF-κB. (**F**) The p38 and NF-κB inhibitors prevent hypoxia-induced TF activity. A549 cells treated with SB202190 (10 μM) or Ro 106-9920 (10 μM) were exposed to hypoxia for 24 h, then the TF-dependent factor Xa generation was determined. Data are presented as mean ± SEM (*n* = 3). * = *p* < 0.05, ** = *p* < 0.01. (**G**,**H**) The p38 activator rescues YC-1′s effect on hypoxia-induced TF expression and procoagulant activity. A549 cells were pretreated with YC-1 (50 μM) in the absence or presence of anisomycin (0.1 μM) and exposed to hypoxia for 24 h. The protein levels (**G**) and activity (**H**) of TF were determined by Western blotting and TF-dependent factor Xa generation, respectively. Data are presented as mean ± SEM (*n* = 3). ** = *p* < 0.01, *** = *p* < 0.001.

MAPKs (ERK, JNK, and p38), as well as Akt, were reported as the upstream regulators of NF-κB [12,13,14]. Therefore, we would like to investigate the role of these protein kinases in hypoxia-induced TF expression. A549 cancer cells were pretreated with either U0126 (ERK inhibitor), SP600125 (JNK inhibitor), SB202190 (p38 inhibitor), or wortmannin (PI3K inhibitor) and then exposed to hypoxic conditions. Figure 6A shows that hypoxia-induced TF expression was abolished by either U0126 or SB202190. In contrast, wortmannin and SP600125 showed slight inhibition and enhancement of TF expression, respectively.

Therefore, we next studied if ERK and p38 were involved in YC-1′s actions. In response to hypoxia, p38 phosphorylation was markedly increased in A549 cancer cells, and was inhibited by YC-1 pretreatment (Figure 6B). The activation of p38 was further confirmed by the appearance of phosphorylation of mitogen-activated protein kinase-activated protein kinase 2 (MAPKAPK2), a specific substrate of p38. Both YC-1 and SB202190 were able to prevent hypoxia-induced increase in phospho-MAPKAPK2 (Figure 6C). In contrast to p38, there was no significant difference in ERK phosphorylation between normoxic and hypoxic conditions, and YC-1 did not reduce the levels of phospho-ERK (Figure 6D). The p38 inhibitor SB202190 mimicked YC-1′s effect to suppressed hypoxia-induced NF-κB phosphorylation (Figure 6E). Additionally, both SB202190 and Ro 106-9920 reduced hypoxia-induced TF activity to the similar levels (Figure 6F). On the other hand, anisomycin, an activator of p38 (Figure S4), reversed YC-1′s effect on inhibiting hypoxia-induced expression and activity of TF (Figure 6G,H).

## 3. Discussion

TF is the major procoagulant expressed by cancer cells and is responsible for the hypercoagulability in cancer patients. The expression of TF in cancer cells can be enhanced under hypoxic conditions that prevail in solid tumors. In this study, we have demonstrated that YC-1, a potential hypoxia-targeted agent, inhibited tumor-associated TF in both protein and mRNA levels in hypoxic cancer cells. YC-1 inhibition of TF expression was associated with a decrease in cell-surface TF activity. The procoagulant activity of hypoxic A549 cancer cells, as indicated by plasma clotting time, was also suppressed by YC-1. It is worth noting that YC-1 had no significant effect on TF expression in A549, MDA-MB-231, and Ca9-22 cells under normoxic conditions, regardless of their basal levels of TF. This indicates that YC-1 inhibition of TF expression is specific for hypoxic responses.

Besides induction of blood coagulation, TF-expressed cancer cells are able to cause platelet aggregation by generation of thrombin, which is also a potent platelet stimulator. Cancer-induced platelet aggregation (CIPA) can lead to arterial thrombosis, such as ischemic stroke and myocardial infarction [33]. In addition, CIPA protects cancer cells from the immune attack of natural killer cells and enhances cancer cells adhesion to and subsequent extravasation through vascular endothelium [34]. Furthermore, cancer-associated platelets facilitate tumor colonization at metastatic sites [35]. CIPA thus plays a critical role in cancer metastasis. We show here that YC-1 treatment results in attenuation of hypoxic A549 lung cancer cells-induced platelet aggregation. This effect is likely due to YC-1 inhibition of hypoxia-induced TF rather than its direct antiplatelet activity, since YC-1 was washed out before the platelet aggregation assay. Nevertheless, the inhibitory effect of YC-1 on CIPA, either directly or indirectly, may be beneficial for preventing cancer-associated arterial thrombosis and cancer metastasis.

HIF-1α is the major mediator of hypoxic responses in cells; however, its role in the regulation of hypoxia-induced TF is controversial. Previous studies showed that HIF-1α knockdown enhanced hypoxia-induced TF expression in human glioblastoma cells, endothelial cells, and podocytes [32,36], suggesting that HIF-1α may act to prevent excessive expression of TF in response to hypoxic conditions. On the other hand, HIF-1α knockdown had an opposite effect in mouse lung and mammary tumor cells [37]. Therefore, the effect of HIF-1α on TF expression may be dependent on species or cell types. In the present study, neither knockdown of HIF-1α by siRNA nor inhibition of HIF-1α by a chemical inhibitor prevented hypoxia-induced TF, indicating that YC-1′s anti-TF effect is unlikely through HIF-1α inhibition.

In addition to HIF-1α, sGC is also a known target of YC-1. Previous studies have reported that liposaccharide-induced TF expression in human monocytes and umbilical vein endothelial cells was negatively regulated by sGC activators [38]. Here we show that in hypoxic A549 cells, YC-1 and another sGC activator BAY 41-2272 stimulated the sGC/cGMP/PKG pathway and inhibited TF expression, suggesting that sGC activation may contribute to the anti-TF effect of both compounds. However, in contrast to BAY 41-2272, which is a more potent and specific sGC activator [31], YC-1′s actions on TF could not be reversed by the sGC inhibitor ODQ, indicating that sGC-independent mechanisms may be involved.

Transcription factors SP1, EGR1, and NF-κB participate TF gene transcription in response to various stimuli, such as lipopolysaccharides (LPS), tumor necrosis factor (TNF), phorbol 12-myristate 13-acetate (PMA), epidermal growth factor (EGF), and hypoxia [39,40]. In particular, EGR1 and NF-κB have been reported to be involved in the regulation of hypoxia-induced responses [36,41]. Here we show that YC-1 prevented hypoxia-induced NF-κB activation and nuclear translocation. In contrast, the nuclear translocation of EGR1 was even enhanced by YC-1, though the cause is unclear at present. These results suggest that inhibition of NF-κB by YC-1 may contribute to its inhibitory effect on hypoxia-induced TF, and this was further supported by the similar effect of the NF-κB inhibitor Ro 106-9920.

The p38 MAPK is known to regulate the NF-κB pathway. The transcriptional activity of NF-κB can be enhanced by p38 through Mitogen- and stress-activated protein kinase-1 (MSK1)-mediated p65 phosphorylation [42]. Furthermore, p38-activated MAPKAPK2 is required for the maintenance of DNA-bound NF-κB [43]. Hypoxia was reported to be a stimulus for p38 activation in many kinds of cells, including lung cancer cells [44,45,46]. In endothelial cells, NF-κB activation in response to hypoxia is enhanced by a p38, but not HIF-1α, -dependent manner [47]. In the present study, the p38/MAPKAPK2 pathway is activated by hypoxia in A549 cancer cells. Inhibiting p38 by SB202190 suppressed hypoxia-activated NF-κB, and reduced TF expression and activity to the similar levels as treatment of Ro 106-9920, suggesting that p38 acts as a positive regulator of NF-κB-mediated TF expression in hypoxic A549 cancer cells. In addition, stimulation of p38 by a known activator, anisomycin [48], reverses YC-1′s effect on hypoxia-induced TF, further supporting that the inhibition of hypoxia-caused p38/NF-κB activation is an underlying mechanism by which YC-1 prevents TF expression.

In summary, we have demonstrated that YC-1 selectively prevents hypoxia-induced TF expression without affecting the basal TF expression under normoxic conditions. This effect is associated with reduced TF procoagulant activity as well as TF-mediated platelet aggregation and unrelated to HIF-1α inhibition. The mode of action of YC-1 is distinct from current anticoagulants that systemically inhibit blood coagulation. This study thus may shed a light on the potential treatment for tumor-associated thrombosis.

## 4. Materials and Methods

### 4.1. Reagents

3-(5′-hydroxymethyl-2′-furyl)-1-benzylindazole (YC-1), sGC inhibitor ODQ, non-competitive selective PDE inhibitor IBMX, and 3-(4,5-dimethylthiazol-2-yl)-2,5-diphenyltetrazolium bromide (MTT) were purchased from Sigma-Aldrich (St. Louis, MO, USA). The PKA inhibitor H89 were purchased from Enzo Life Sciences (Farmingdale, NY, USA). The sGC activator BAY 41-2272 and NF-κB inhibitor Ro 106-9920 were purchased from Santa Cruz Biotechnology (Santa Cruz, CA, USA). The extracellular regulated MAP kinase (ERK) inhibitor U0126, p38 MAP kinases inhibitor SB202190, c-Jun N-terminal kinases (JNK) inhibitor SP600125, HIF-1α inhibitor CAY10585, p38 activator anisomycin, and phosphatidylinositol-3-kinases (PI3K) inhibitor wortmannin were purchased from Cayman Chemical (Ann Arbor, MI, USA).

### 4.2. Cell Culture and Hypoxia Incubation

Human non-small cell lung cancer A549 cells and human triple negative breast cancer cell line MDA-MB-231 cells were purchased from Bioresource Collection and Research Center (BCRC, Taiwan). The human oral squamous cell carcinoma cell line Ca9-22 was kindly provided by Prof. Jeff Yi-Fu Chen (Kaohsiung Medical University). The cells were maintained in Dulbecco’s Modified Eagle’s Medium (DMEM)/F12 (Gibco, Thermo Fisher Scientific, Waltham, MA, USA) supplemented with 10% fetal bovine serum (FBS) (Gibco, Thermo Fisher Scientific, Waltham, MA, USA) and 1% penicillin/streptomycin (Invitrogen, Thermo Fisher Scientific, Waltham, MA, USA), and incubated in a humidified incubator at 37 °C with 5% CO_2_. For executing hypoxia experiments, cells were incubated in modular incubator chamber (Billups-Rothenberg, Inc., Del Mar, CA, USA) and flushed with 1% O_2_, 5% CO_2_, and 94% N_2_ to mimic hypoxia.

### 4.3. MTT Assay

Cancer cells were seeded in a 96-well plate (1 × 10^4^ cells/well) and treated with drugs in 5% CO_2_ at 37 °C for 24 h in normoxic or hypoxic conditions. At the end, culture medium was removed and 100 µL of MTT solution (0.5 mg/mL) was added to each well. Cells were further incubated at 37 °C for 2 h. The formazan crystals were solubilized with DMSO and the absorbance was read at 550 nm.

### 4.4. Cell Surface TF Activity Assay

Cancer cells were seeded in a 96-well plate (5 × 10^3^ cells/well) and incubated overnight in 5% CO_2_ at 37 °C. Cells were pretreated with drugs for 1 h, followed by 24 h in hypoxic or normoxic conditions. After removal of culture medium, cells were washed twice with PBS, and incubated with human factor VIIa 10 nM (Haematologic Technologies, Inc., Essex Junction, VT, USA) and human factor X 175 nM (Haematologic Technologies, Inc., USA) in Tris-buffered saline (TBS, containing 5% BSA and 25 mM CaCl_2_) at 37 °C for 10 min. The reaction was terminated by adding the stop solution (TBS with EDTA 25 mM). Then, Spectrozyme factor Xa substrate (1 mM, Sekisui Diagnostics, Lexington, MA, USA) was added, and the absorbance was read in kinetic at 405 nm. The end point of the absorbance linear curve was recorded, and the results of TF activity were normalized to cell viability using MTT assay.

### 4.5. Measurement of Cancer-Induced Platelet Aggregation

Human blood was isolated from healthy human volunteers and anticoagulated with acid citrate dextrose (ACD). This study was approved by the Institutional Review Board of Kaohsiung Medical University, and informed consent was acquired from all volunteers. The preparation of platelet suspension was described as previously experimental [30]. Platelet suspension was prepared to 3 × 10^8^ platelets/mL in Tyrode’s solution (2 mM CaCl_2_, 1 mM MgCl_2_, 11.1 mM glucose, and 0.35% (*w*/*v*) bovine serum albumin). Cancer cells treated with drugs under normoxic or hypoxic conditions were harvested (1 × 10^5^ cells/mL) and mixed with platelet suspension (3 × 10^8^ platelets/mL). Platelet aggregation was triggered by adding human plasma (0.25%) and measured by using turbidimetric aggregometer (Chrono-Log Co., Havertown, PA, USA) under stirring conditions (1200 rpm) at 37 °C.

### 4.6. Tilt Tube Plasma Clotting Assay

Plasma clot induced by cancer cells was measured as previously described [13]. In brief, cancer cells treated with drugs under hypoxic or normoxic conditions were harvested and finally resuspended in PBS at a concentration of 2.5 × 10^5^ cells/mL. 200 μL of cancer cell suspension was mixed with an equal volume of human platelet-poor plasma in a test tube, then 200 μL of CaCl_2_ solution (25 mM in normal saline) was added to trigger plasma clot at 37 °C. Plasma clotting was observed visually and checked every 5 s. Recording the clotting time when plasma formed a semisolid gel that did not flow during tube tilting.

### 4.7. RNA Interference

Silencer^®^ Select HIF-1α siRNA were purchased from Ambion (Thermo Fisher Scientific, USA), and transfected by using Lipofectamine^®^ 2000 Transfection Reagent (Thermo Fisher Scientific, USA). Cells were treated with siRNA in an optimal concentration (50 nM) for 24 h, then the culture medium was replaced with fresh medium. Transfected cells were further incubated for 48 h before exposing to hypoxia or normoxia for the indicated time. Silencer^®^ Select Negative Control siRNA was used as negative control.

### 4.8. Reverse Transcription Real-Time Polymerase Chain Reaction (PCR)

Total mRNA was isolated by GeneMark Total RNA Miniprep Purification Kit (GMbiolab Co, Ltd., Taiwan). The mRNA templates were converted into a complementary DNA (cDNA) by using a High-Capacity cDNA Reverse Transcription Kit (Applied Biosystems™, Foster City, CA, USA). KAPA SYBR^®^ FAST qPCR Master Mix was mixed well with the cDNA samples and primers. The polymerase chain reaction was carried out and analyzed by using Applied Biosystems™ StepOne™ Real-Time PCR System. Every single experiment was performed in triplicates. The fold change in gene expression levels was calculated with normalization to β-actin values by using 2^−ΔΔCt^ comparative cycle threshold method. All of the primer sequences used for this study are listed in Table 1.

### 4.9. Western Blot Analysis

Cells were harvested by cell scraper and centrifuged for 15 min (300 g at 4 °C). Then, cells were sonicated and lysed with lysis buffer (50 mM Tris-HCl pH = 7.4, 150 mM NaCl, 1 mM EDTA, 1 mM EGTA, 1% Triton X-100) containing protease inhibitor and phosphatase inhibitor (Roche Applied Science, Penzberg, Germany) for 15 min on ice. Cell lysates were centrifuged for 15 min (16,000*g* at 4 °C) and the supernatant was collected. The concentrations of protein samples were determined by using the Bio-Rad Protein Assay Kit II (Bio-Rad, #5000002, Hercules, CA, USA). Equal amounts of protein were separated by SDS-PAGE and transferred to nitrocellulose membranes. After blocking with 5% non-fat milk in TBST (tween-20, TBS) for 1 h, the membrane was washed four times with TBST, each time for 5 min, and incubated with primary antibody at 4 °C overnight. After washing four times with TBST, the membrane was incubated with horseradish peroxidase (HRP)-labeled secondary antibody for 1 h at room temperature. The images of chemiluminescence signal were captured by Luminescence/Fluorescence Imaging System LAS-4000 (Fujifilm, Tokyo, Japan). Antibodies against various proteins were listed as follows: tissue factor (#4509) antibody was purchased from Sekisui Diagnostica (USA). HIF-1α (#610958) antibody was purchased from BD Biosciences, EGR1 (#sc-189), ERK (#sc-94), phospho-ERK T204 (#sc-7383), Sp1 transcription factor (SP1) (#sc-59), and β-Actin (#sc-8432) antibody were purchased from Santa Cruz Biotechnology (Santa Cruz, CA, USA). Phospho-p38 Thr180/Tyr182 (#9216), p38 (#9212), NF-кB p65 (#4764), phospho-NF-κB p65 Ser536 (#3033), IкBα (#4814), phospho-IкBα Ser32 (#2859), phospho-VASP Ser157 (#3111), phospho-VASP Ser239 (#3114), MAPKAPK2 (#3042), phospho-MAPKAPK2 Thr334 (#3007), GAPDH (#2118), and α-Tubulin (#2144) antibodies were purchased from Cell Signaling Technology Inc. (Danvers, MA, USA). VASP (#V3390) antibody was purchased from Sigma-Aldrich (USA). ORC2 (#M055-3) antibody was purchased from MBL International Corporation (Moburn, MA, USA).

### 4.10. Statistical Analysis

All data were processed and analyzed using Prism4^®^ (Graphpad Software, Inc., San Diego, CA, USA) and performed as mean ± SEM of the indicated number for separate experiments. Significance was estimated by using one-way analysis of variance (ANOVA) tests. Asterisks in the figures indicate significance (* *p* < 0.05, ** *p* < 0.01, *** *p* < 0.001).

## Figures and Tables

**Table 1:** Specific primer sequences used for real-time PCR analysis.

Gene	Forward	Reverse
TF	5′-GCCAGGAGAAAGGGGAAT-3′	5′-CAGTGCAATATAGCATTTGCAGTAGC-3′
VEGF	5′-TCGGGCCTCCGAAACCATGA-3′	5′-CCTGGTGAGAGATCTGGTTC-3′
β-actin	5′-TCACCCACACTGTGCCCATCTACGA-3′	5′-CAGCGGAACCGCTCATTGCCAATGG-3′

## Supplementary Materials

The following are available online at http://www.mdpi.com/1422-0067/20/2/244/s1.
