# Supplementary material for: YC-1 Prevents Tumor-Associated Tissue Factor Expression and Procoagulant Activity in Hypoxic Conditions by Inhibiting p38/NF-κB Signaling Pathway
Source: Int J Mol Sci. 2019 Jan 9;20(2):244. doi: 10.3390/ijms20020244 (PMC6359014; doi:10.3390/ijms20020244)
Supplement: Supplementary file 1 [file ijms-20-00244-s001.pdf]

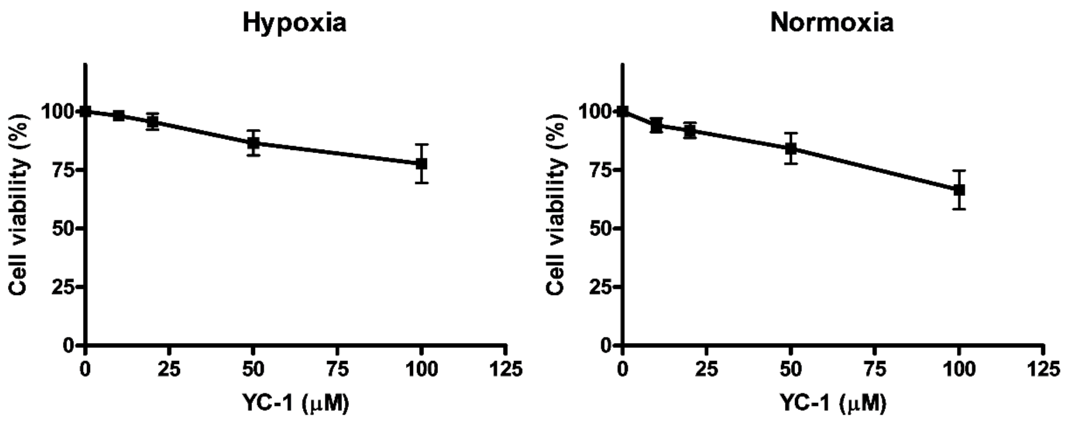

Figure S1 Effect of YC-1 on cell viability of A549 cells. A549 cells were pretreated with YC-1 for 1 hour and incubated under hypoxic or normoxic conditions for 24 hours. The effect of YC-1 on cell viability was determined by MTT assay.

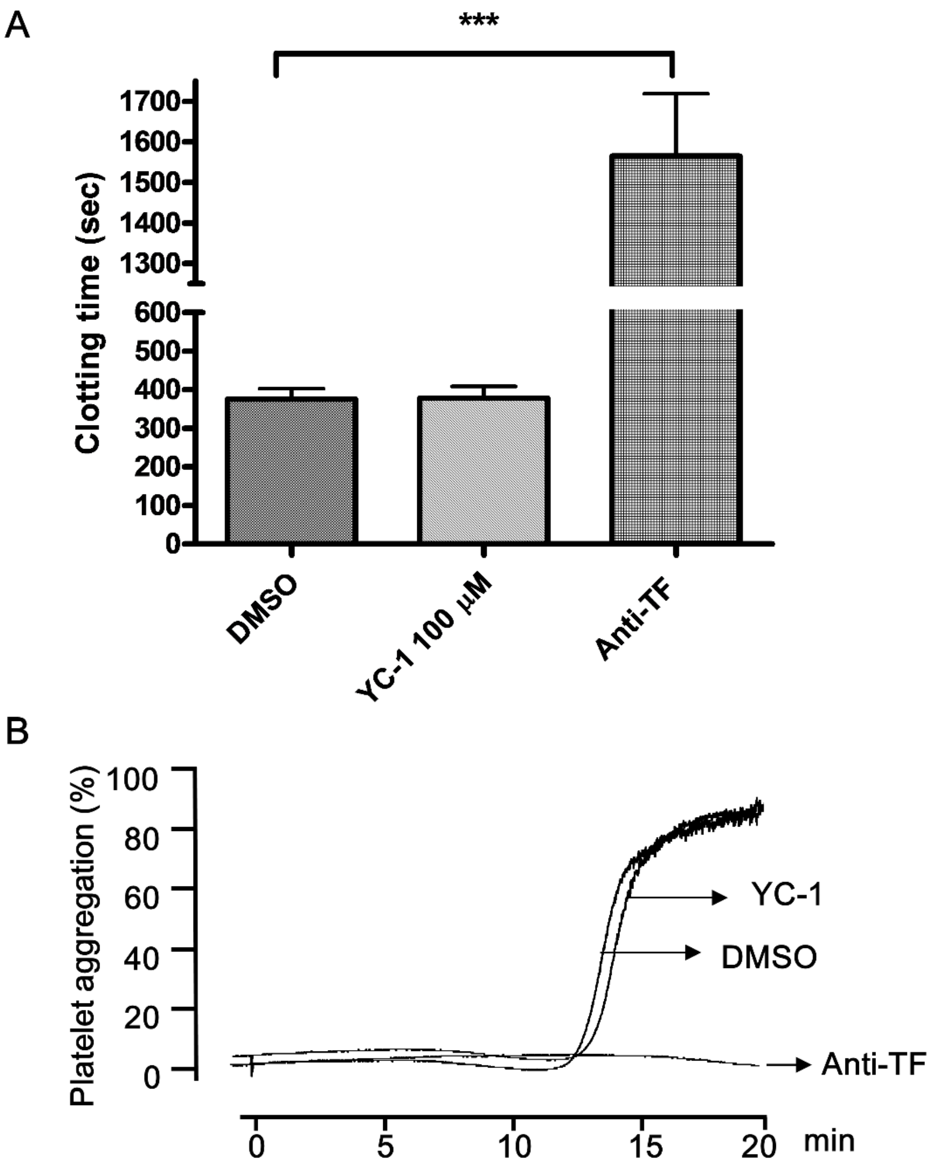

Figure S2 Effects of YC-1 on A549 cell-induced plasma clotting and platelet aggregation in normoxic conditions. A549 cells were treated with DMSO or YC-1 (100 μM) for 24 hours in normoxia. Cancer cell-induced plasma clotting (A) and platelet aggregation (B) were determined as described in Methods. Anti-TF antibody (20 μg/ml) was used as positive control. All results are presented as mean ± SEM (n = 3). *** = *P* <0.001.

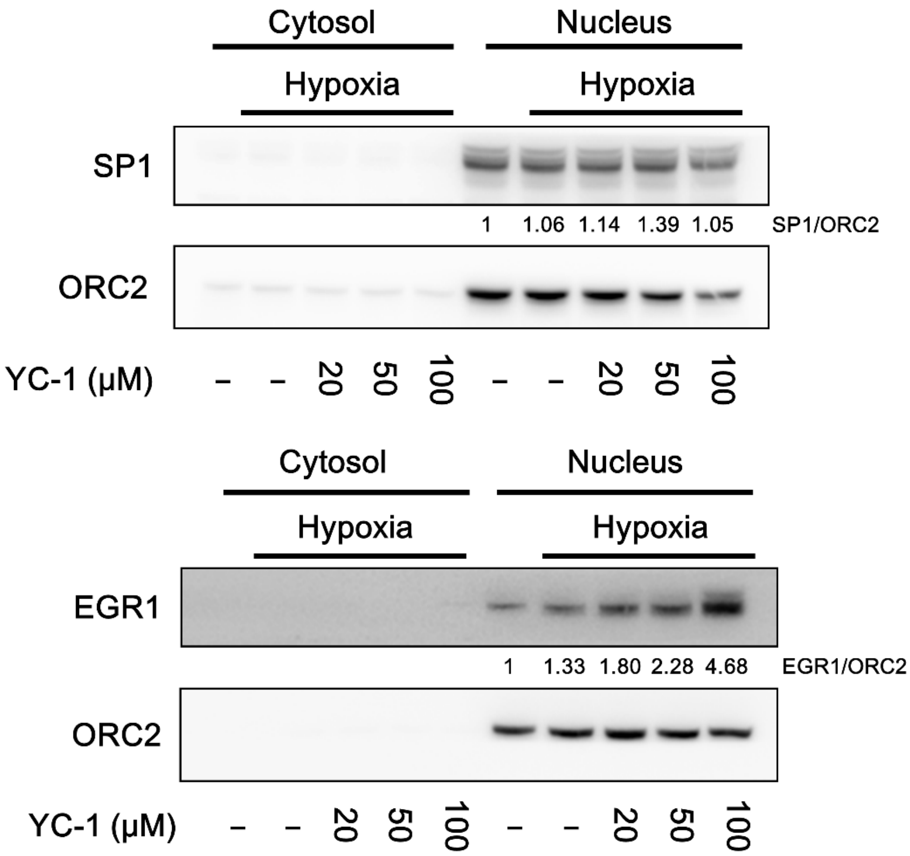

Figure S3 Effects of YC-1 on nuclear translocation of Sp1 and Egr1 in A549 cells. A549 cells were pretreated with YC-1 for 1hour and exposed to hypoxia for 1 hour. Protein levels of SP1, and Egr-1, and in the nuclear and cytoplasmic fractions were determined by Western blot.

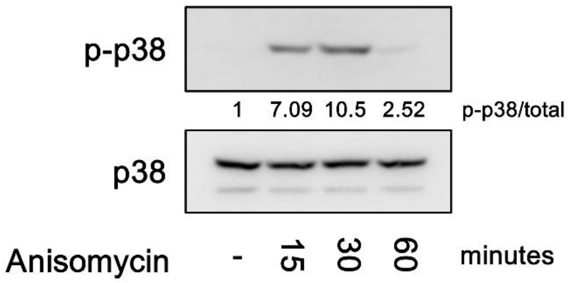

Figure S4 p38 activation by p38 activator in A549 cells. Cells were treated with anisomycin (0.1 μM) for indicated times. Protein expression were determined by Western blot.
